# Highlights from the 13th St Gallen International Breast Cancer Conference 2013. Access to innovation for patients with breast cancer: how to speed it up?

**DOI:** 10.3332/ecancer.2013.299

**Published:** 2013-03-26

**Authors:** Giuseppe Curigliano, Carmen Criscitiello, Fabrice Andrè, Marco Colleoni, Angelo Di Leo

**Affiliations:** 1 Division of Early Drug Development, European Institute of Oncology, Milan, Italy; 2 Department of Medical Oncology, Institut Gustave Roussy, Villejuif, France; 3 Division of Medical Senology, European Institute of Oncology, Milan, Italy; 4 Department of Oncology, Hospital of Prato, Istituto Toscano Tumori, Prato, Italy

**Keywords:** 13th St Gallen Consensus Conference 2013, Early Breast Cancer

## Abstract

The recognition that early breast cancer is a spectrum of diseases each requiring a specific systemic therapy guided the 13th St Gallen International Breast Cancer Consensus Conference [1]. The meeting assembled 3600 participants from nearly 90 countries worldwide. Educational content has been centred on the primary and multidisciplinary treatment approach of early breast cancer. The meeting culminated on the final day, with the St Gallen Breast Cancer Treatment Consensus, established by 40–50 of the world’s most experienced opinion leaders in the field of breast cancer treatment. The major issue that arose during the consensus conference was the increasing gap between what is theoretically feasible in patient risk stratification, in treatment, and in daily practice management. We need to find new paths to access innovations to clinical research and daily practice. To ensure that continued innovation meets the needs of patients, the therapeutic alliance between patients and academic-led research should to be extended to include relevant pharmaceutical companies and drug regulators with a unique effort to bring innovation into clinical practice. We need to bring together major players from the world of breast cancer research to map out a coordinated strategy on an international scale, to address the disease fragmentation, to share financial resources, and to integrate scientific data. The final goal will be to improve access to an affordable, best standard of care for all patients in each country.

## Introduction

The primary aim of the 2013 St Gallen Consensus conference was to provide rational recommendations for personalising the approach to treatment of women with early breast cancer. Major issues that have been highlighted included: (1) tumour biology to determine responsiveness to various treatments, (2) tumour extent to estimate the level of benefit when justifying treatment for the individual patient, and (3) estimates of the risks of therapy and patient preference to define preferred management. The areas of controversy discussed during the meeting were: (1) surgery of the primary; (2) surgery of the axilla; (3) radiation—partial breast, post-mastectomy, nodal areas, advanced technologies; (4) pathology; (5) multigene signatures; (6) stroma—extracellular matrix and prognosis; (7) endocrine therapies—ovarian suppression, tamoxifen, aromatase inhibitors; (8) chemotherapies—luminal A, duration, regimen; (9) anti-HER2 therapies—combination, duration; (10) neo-adjuvant systemic therapy; (11) bisphosphonates—anti-tumour effects; and (12) follow-up after early breast cancer. Here we present a conference report that will highlight some of the controversial areas discussed during this outstanding meeting.

## News since St Gallen 2011

Dr Jose Baselga from New York, USA, opened the scientific session with an outstanding and comprehensive talk on the role of PIK3CA inhibitors in subtypes of breast cancer. The driver role of this pathway is emerging not only for endocrine responsive disease but also for triple-negative breast cancer (TNBC). Dr Angelo Di Leo updated evidence from the Oxford overview. This latter meta-analysis demonstrated the clear benefit of adjuvant chemotherapy over no chemotherapy; improved outcomes when using anthracycline-based therapies compared with CMF; and a small but significant improvement in disease-free, breast cancer-specific, and overall survival with the addition of taxanes to anthracyclines. In contrast to the increasingly recognised importance of tumour biology in treatment response, all subgroups were shown to benefit from chemotherapy Although absolute benefits varied relative to tumour stage, there were proportional benefits, roughly equivalent across subgroups, irrespective of tumour size, nodal status, grade or ER status. The utility of large-scale meta-analyses as provided by the overview is potentially contradictory to our growing understanding of breast cancer as multiple diseases, rather than a single disease entity. Increased importance of tumour biology on systemic therapy response is now recognised; however, this cannot be readily assessed within the overview, since the included studies have not incorporated data on characteristics such as proliferation index, intrinsic subtype, and function of DNA repair mechanisms. As we move towards individualised therapies, Dr Di Leo concluded that the overview is only likely to remain relevant if it can be adapted to address additional or alternate questions. Rather than assessing whether all patients benefit from a particular intervention, assessment should be directed to whether particular subgroups benefit from specific therapies based on the tumour characteristics. Dr WD Foulkes from Canada discussed emerging targets for TNBC. There is evidence to suggest that different subtypes of TNBCs are likely to respond to different therapeutic strategies. While those with dysfunctional homologous recombination DNA repair defects (e.g. BRCA1/2 mutant) have been shown to derive benefit from platinum salts and PARP inhibitors, other subtypes may benefit from PIK3CA/mTOR inhibitors (e.g. TNBCs harbouring PIK3CA mutations and/or PTEN loss of function) or anti-androgen receptor therapies (e.g. luminal androgen receptor TNBCs). The clinical and biological significance of the mesenchymal, mesenchymal stem-like, and claudin-low tumours remains to be fully elucidated. It is plausible, in the future, that combinatorial therapies targeted to more than one pathway will be more successful than targeting individual pathways, given the degree of intra-tumour genetic heterogeneity found in TNBCs. Dr K Osborne delivered an outstanding lecture on mechanisms of resistance to endocrine therapy. Hyperactive PI3K signalling is associated with a more aggressive subtype of ER-positive breast cancer and may contribute to resistance to endocrine therapy. High PI3K pathway activation correlates with the luminal B subtype of breast cancer and is associated with lower ER expression. We developed a laboratory model to study the effects of PI3K pathway activation on endocrine therapy resistance and used this model to study a variety of pathway inhibitors alone and in combination to obtain clues as to which combination of inhibitors might be optimal in the clinic.

## Clinical application of genomic sequencing technologies

Dr MJC Ellis from St Louis, MO, USA, inspired the audience by discussing the application of high-throughput next-generation sequencing technologies to personalised treatment for patients with breast cancer. Wide-genome analysis approaches will produce a more complete picture of the biology present in each individual tumour. The combined knowledge base provided by a recent next-generation sequencing studies is unprecedented, but it will take many years before all the therapeutic hypotheses raised by this vast data repository will be addressed. New therapeutic road maps are emerging and the opportunities in luminal-type breast cancer are particularly compelling. Dr Ellis concluded that a new treatment paradigm is therefore evolving, whereby deep genomic analysis will drive treatment decisions based on a pharmacopoeia of cell-type and pathway-matched therapies. Dr DF Hayes from the USA, methodologically dissected potential pitfalls of ‘omics’ approach to individualised therapy. Once an omics-based test is analytically developed that has clinical and/or biologic validity, the investigative team might follow specific pathways to establish clinical utility and therefore acceptance by regulatory and guideline bodies: that is, prospective retrospective studies or prospective studies in which the omics-based test is the primary objective of the trial itself. These pathways are rigorous and therefore not easily accomplished. Dr Hayes concluded that if we are to apply these tests to direct management of our patients, we must approach the science of biomarker development with the same rigour that is used for therapeutic agent assessment. Dr P Bertheau from Paris, France, discussed p53-mediated response to chemotherapy in breast cancer subtypes. In ER+ TP53 wild-type (WT) breast cancer, ER-induced inhibition of p53 apoptotic response would lead preferentially to tumour cell senescence and subsequent resistance to treatment. Conversely, in ER-TP53-mutated breast cancer, especially in those having lost both TP53 alleles, accumulation of genetic abnormalities would lead to a mitotic catastrophe and subsequent better response. In view of these recent results, p53 impact in breast cancer should be reconsidered. Dr CM Perou from the University of North Carolina, NC, USA, delivered a ‘hot topic’ lecture on prediction of treatment response by gene expression profiling assays.

## Genetics, prevention and metabolism

Dr RT Chlebowsky from Los Angeles, CA, USA, discussed the impact of nutrition and physical activity on breast cancer incidence and recurrence. While the definitive impact of nutrition and clinical activity on clinical breast cancer outcomes awaits randomised clinical trial results, available evidence suggests such interventions could reasonably be considered for incorporation in current breast cancer management. Dr M Dowsett from London, UK, reported on sex hormones and breast cancer risk. The use of highly sensitive assays revealed that the association between BMI and plasma oestrogen assays persists in patients on aromatase inhibitors, and that measurable increments in plasma oestrogen levels occur with some vaginal oestrogen preparations that are of concern in relation to the treatment efficacy. Dr Judy Garber from Boston, MA, USA, described the potential clinical implications of germ cell-line mutations in patients with hereditary breast cancer. Despite promising results in terms of pathological complete response induced by cisplatin monotherapy in BRCA-mutated patients, we do not have sufficient evidence to incorporate it as a standard of care in daily practice treatment of patients with TN BRCA-mutated breast cancer. Dr P Goodwin from Toronto, Canada, gave an outstanding lecture on the impact of metabolism on breast cancer outcome. There is an urgent need for adequately powered adjuvant weight loss studies to examine the impact of effective weight loss interventions on BC outcomes.

## Stroma and tumour crosstalk

Dr F Bertolini from Milan, Italy, delivered a discussion on crosstalk between white adipose tissue (WAT) and breast cancer cells. His studies indicate that human WAT transplant can promote breast cancer angiogenesis and growth and metastases through complementary mechanisms. Dr RE Coleman from Sheffield, UK, addressed crosstalk of the microenvironment with breast bone metastases. Bone metastases result from the interactions between cancer cells in the bone marrow microenvironment, haematopoietic stem cells and normal bone cells. Our understanding of these complex processes has increased in recent years and has provided an alternative strategy for the prevention of metastasis through the use of bone-targeted treatments to modify the bone marrow microenvironment. Improvements in both diseasefree survival (DFS) and overall survival in women with early breast cancer have been demonstrated in several large randomised adjuvant trials of intravenous zoledronic acid and oral clodronate. Dr R Kerbel from Toronto, Canada, addressed the critical issue of antiangiogenic therapy in breast cancer. The results of three bevacizumab-based phase III adjuvant trials (also involving combination with chemotherapy) – two in colorectal cancer (CO8 and AVANT) and one in breast cancer (BEATRICE) – have shown no benefit in DFS times, or even a trend to a worse survival outcome (AVANT). Taken together, these results suggest the potential improved value of using postsurgical early or advanced metastatic disease models. In this regard, Dr Kerbel’s group have found that several different low-dose metronomic chemotherapy regimens, for example, cyclophosphamide plus the 5-FU oral prodrug UFT or oral topotecan plus an antiangiogenic drug (e.g. pazopanib) have marked efficacy when used to treat advanced metastatic breast cancer. Dr T. Oskarsson from Heidelberg, Germany, addressed the role of extracellular matrix player in breast cancer progression and metastasis.

## Screening, diagnosis and pathological assessment

Dr DA Berry from Houston, TX, USA delivered a provocative, outstanding talk on the role of breast cancer screening. He chronicled the evidence and controversies regarding mammographic screening, including attempts to assess the relative contributions of screening and therapy in the substantial decrease in breast cancer mortality that has been observed in many countries over the last 20 years or so. He emphasised the trade-off between harm and benefits depending on the woman’s age and other risk factors. He also discussed ways for communicating the associated risks to women who have to decide whether screening (and what screening strategy) is right for them. Extensive discussion with the audience followed his presentation. Dr SH Heywang-Kobrunner from Munchen, Germany, addressed magnetic resonance imaging (MRI) in breast cancer diagnosis. MRI is quite helpful as an additional diagnostic tool for specific questions, such as the search for the primary tumour, diagnostic problems after silicon implant, breast conservation or scarring and differentiation of abnormalities detected on one mammographic view. Overall, MRI certainly represents an important further step for improving breast imaging. Research concerning its diagnostic value and further developments is ongoing. Dr L Pusztai from Yale University, CT, USA, addressed influence of genomics in adjuvant treatment choice. Genomics fundamentally altered our perception of breast cancer that is now considered as at least four biologically distinct diseases that require different therapeutic approaches and clinical trials. A major issue will be to incorporate genomics in daily clinical practice. As discussed in the consensus conference, many countries do not have access to ‘omics’ to predict prognosis and potential responsiveness to standard adjuvant. This is a major issue when selecting optimal adjuvant treatment in patients with early breast cancer. Dr G Viale from Milan, Italy, discussed the importance of characterisation of residual disease after neoadjuvant chemotherapy. The assessment of the extent of residual disease after neoadjuvant chemotherapy and its biological characterisation are important prognostic parameters and valuable data to inform the choice of subsequent systemic interventions. Indeed, pathologic complete response (pCR) is associated with a more favourable disease-free and overall survival than observed for patients with residual disease. In general, the biological characteristics (especially oestrogen receptor and HER2 status) of the tumours remain the same before and after neo-adjuvant chemotherapy, but in some instances, discrepancies will be encountered. In these cases, it may be difficult to sort out if the changes actually represent laboratory errors in testing, a consequence of intratumour heterogeneity in the expression of the markers or true biological effects of the systemic therapy. There is a substantial need for harmonisation of the way pathologists are handling, examining and reporting breast cancer after neoadjuvant chemotherapy.

## Experimental diagnostics and therapeutics

This session opened a window to the potential future of personalised medicine for patients with breast cancer. Dr F Andrè from Paris, France, addressed the role of molecular predictors for risk stratification in order to tailor adjuvant therapies. Identification of patient subgroup could lead to the decrease of the indications of adjuvant chemotherapy, based on the identification of a subpopulation of patients with very low risk of relapse. The identification of such a group could be associated with the potential risk of not treating patients who could potentially derive benefit from adjuvant chemotherapy. In order to illustrate the ongoing research on this topic, he discussed the potential use of Ki67, tumour-infiltrating lymphocytes, and residual disease for treatment decision. Dr C Sotiriou from Brussels, Belgium, assessed the predictive and prognostic role of immune signatures in patients with breast cancer. His group showed that an immune response module, the STAT1 module, was associated with survival in TN and HER2+ patients, and in the same breast cancer subtypes, other investigators showed that the overexpression of immune-related genes was able to identify subgroups of patients with a better prognosis. Evidence also indicates that activation of the immune signatures could mediate the antitumour effects of several anticancer drugs. Dr G Curigliano from Milan, Italy, highlighted the potential role of immunotherapy in patients with early breast cancer. Cancer takes advantage of the ability to hide from the immune system by exploiting a series of immune escape mechanisms that were developed to avoid autoimmunity (mechanisms of tolerance). Among these mechanisms are the hijacking of immune-cell intrinsic checkpoints that are induced on T-cell activation. The blockade of one of these checkpoints, cytotoxic T-lymphocyte-associated antigen-4 (CTLA-4) or the programmed death-1 (PD-1) receptor, recently provided the first evidence of activity of an immune-modulation approach in the treatment of a solid tumour. The future frontier in the treatment of cancer requires identification of potential targets in order to personalise therapies. The immune system remembers what it targets, so once the system is correctly activated, it may mediate a durable tumour response.

## Advances in surgical management of early breast cancer

Dr V Galimberti from Milan, Italy, discussed the role of sentinel node (SN) biopsy in the management of patients with operable breast cancer. It is now clear that the best way to handle positive sentinel nodes is to do nothing further to the axilla, provided patients receive breast irradiation and systemic adjuvant treatment. In the future, we may not need to use any surgical method to determine whether the axilla is involved, and even if the axilla is positive, axillary dissection today appears as overtreatment in most cases. Dr M Hamdi from Brussels, Belgium, addressed reconstructive and oncoplastic surgery. The pedicled perforator flap is a new concept in breast surgery. Besides its functional benefit due to the minimal donor site morbidity, it gives advantages in flap shaping and consequently better aesthetic results and higher patient satisfaction. Dr M Morrow from New York, USA, discussed personalised surgery in the era of breast cancer subtypes. The benefit of systemic therapy on local control may allow the intensity of local therapy (surgery or radiotherapy) to be decreased, and that approaches to optimise local therapy may not be uniform across molecular subtypes of breast cancer. Dr EJT Rutgers from Amsterdam, The Netherlands, discussed potential contraindications to breast conservation. Some clinical situations can increase the risk of relapse and should be extensively discussed with the patient. Breast conservation should be executed with caution in the following settings: very young women (<35 years), or extensive ductal carcinoma *in situ *(heralded by extensive microcalcifications) mounting up to one-quarter of the breast, particularly in women under 40 years of age. Prof WC Wood from Atlanta, GA, USA, addressed the impact of close/positive margins on breast cancer recurrence. The margin of normal tissue beyond the primary tumour that significantly reduces the risk of local recurrence remains undefined. Sufficient data are available to say that in the era of systemic therapy, excellent radiation therapy techniques, and boost doses when indicating no margin of normal breast tissue beyond the tumour, has been shown to be clearly superior to a layer of cells between the ink and the tumour. However, a larger tumour size and aggressive biology are reason to be less confident that a single layer of cells at the point of histologic study accurately represents a clear margin. As in all medical decisions, wise judgement must integrate all the known factors to reach the best recommendation. There are few circumstances that would warrant a second surgical procedure for a close but clear margin today.

## Areas of controversy and newer radiation approaches

Dr BV Offersen from Denmark discussed regional node radiotherapy. An alternative to axillary lymph-node dissection (ALND) is treating the axilla with nodal radiotherapy (RT) although this treatment is mostly used as adjuvant treatment after ALND in high-risk patients. Few studies have investigated the benefit of nodal RT compared with ALND in SN-positive low-risk patients, and no consensus has yet been reached. Clinical decision making regarding treating the axilla should be based on relevant data, and in this review, studies aimed at deciding whether or not, and also how the axilla should be treated in SN-positive patients will be discussed. Dr J Harris from Boston, USA, discussed hypofractionated radiotherapy as an emerging approach in the field of breast cancer treatment. The majority of patients included in the studies supporting hypofractionated treatment were older (<25% were aged <50), with early-stage invasive ER+ disease treated with BCT. Additionally, few or none of the patients included in most studies were treated with lymph node irradiation (<7%), a lumpectomy cavity radiation boost, or adjuvant chemotherapy (<22%). This favourable subset of patients is also the most eligible for other alternative treatment approaches, such as partial-breast irradiation. Nevertheless, there is now sufficient evidence to recommend hypofractionated whole-breast RT for a substantial percentage of patients. Dr R Orecchia from Milan, Italy, addressed the role of partial breast irradiation (PI). The comparison between the current standard for early-stage breast cancer with early data coming from PBI techniques poses a dilemma as to when preliminary results are sufficiently mature to allow practitioners and patients to consider a new treatment approach as safe, knowing that most data from controlled and/or randomised studies of breast conservation therapy have demonstrated the importance of very long-term data in determining the ultimate efficacy of a treatment. New issues of biology-oriented patient selection need to be validated with independent datasets and a new generation of randomised trials, in order to be more appropriate in specific situations. Dr F Sedlmayer from Salzburg, Austria, discussed breast re-irradiation after breast cancer recurrence. In a highly selected group of patients with local recurrence, partial breast irradiation after second breast cancer surgery is a viable alternative to mastectomy, yielding high breast preservation rates without compromising oncologic safety. Whereas the evidence for brachytherapy is more solid, there is still little information regarding the effectiveness of partial breast irradiation via external beam radiotherapy or novel strategies like intraoperative radiotherapy, which therefore should preferably be investigated within trials.

## Adjuvant systemic treatment for the individual patient

Dr KS Albain from Loyola University, IL, USA, delivered an outstanding lecture on the management of luminal HER2-negative breast cancer, with special attention to bad stage or high-risk disease but with ‘good’ or favourable biology, as well as the opposite scenario of apparently low-risk disease with a ‘bad’ biologic profile. The most challenging clinical scenario is the subgroup of patients with very high risk luminal ER-positive HER2-negative disease (by standard clinical-pathologic features) but who also have ‘favorable’ biology as defined by multigene assays. This group is relatively resistant to standard chemotherapy, and there is insufficient efficacy of the endocrine therapy. In this group, optimising inhibition of cross-talk with other pathways that take over and drive tumour growth – despite the endocrine therapy blockade – is mandatory. Prof. Harold Burstein from Dana Farber Cancer Institute, Boston, MA, USA, addressed the very special case of adjuvant treatment of TNBC. Based on direct comparisons, subset analyses, and considerations of toxicity/tolerability, optimal adjuvant treatment should include sequential anthracycline, cyclophosphamide, and taxane-based therapy. A preferred regimen without anthracyclines should include docetaxel and cyclophosphamide. A preferred regimen without taxanes should be AC or CMF. Dr Gunter von Minckwitz of the German Breast Group highlighted the importance of pCR after neoadjuvant chemotherapy. It has become more evident throughout the last couple of years that pCR in patients with HER2-positive or TNBC shows a high correlation with a low risk of relapse. However, in luminal-type tumour, this correlation is less strong to use this for clinical treatment decisions. This observation has recently been confirmed by an FDA-initiated global meta-analysis on 13,000 patients from neoadjuvant chemotherapy trials. Prof M Piccart from Brussels, Belgium, gave a lecture on HER2-targeted agents. The optimal duration of trastuzumab therapy currently remains one year. The HERA trial showed that two years does not confer additional advantage over one year, which remains the standard of care until results from SOLD, Short-HER and PERSEPHONE consolidate or negate this finding. The PHARE trial (6 versus 12 months of trastuzumab) showed a trend favouring one year of treatment but was not fully conclusive. The phase III RCT HannaH compared the pharmacokinetic profile, efficacy, and safety of the subcutaneous (SC) and intravenous (IV) formulations of trastuzumab. The SC formulation was proven non-inferior to the standard IV, although the incidence of serious adverse events was higher in the SC arm. The authors concluded that SC trastuzumab, administered at a fixed dose of 600 mg over 5 min, could be a valid alternative option, with the potential for human and economic savings in clinical practice. In 1–2 years from now, the first results of dual HER2 targeting with trastuzumab and lapatinib will become available. Professor Ian Smith from Royal Marsden, UK, discussed the topic of follow-up in patients with breast cancer. There are several reasons to consider routine regular follow-up after early breast cancer. These include: (1) to improve outcome and/or outcome of quality of life (QOL) through earlier detection of recurrence, (2) to detect and treat late treatment-related effects, (3) to collect long-term follow-up data for research and audit, and (4) to detect new primaries in higher risk women. The key problem in this field is that although regular follow-up seems self-evident and desirable for many patients, data to support its benefit are largely lacking. Meanwhile standard follow-up should remain clinical assessment and annual mammography. Dr Marco Colleoni from European Institute of Oncology, Milan, Italy, stressed the role of extended adjuvant chemotherapy in special subpopulation of breast cancer patients. Patients treated with sequential chemotherapy had a significant reduction in relapse or death as compared with shorter duration of therapy. Prolonged chemotherapy appeared to be more efficacious in all subgroups including ER-negative disease. The Breast International Group 02–98 trial compared sequential and concurrent administration of doxorubicin and docetaxel. Patients with four or more positive lymph nodes, as well as patients with hormone receptornegative disease showed the largest absolute improvement in five year DFS when the sequential docetaxel arm (doxorubicin, followed by docetaxel, followed by CMF) was compared with the control arms. Dr Nancy Davidson from the University of Pittsburgh, Pittsburgh, PA, USA, discussed the optimal treatment of premenopausal patients with breast cancer. Although systemic therapy is one of the cornerstones of therapy for premenopausal women with early-stage breast cancer, there remain many unknowns regarding its optimal use. Information about the value of ovarian suppression continues to emerge, most recently with the demonstration of excellent outcome with goserelin plus tamoxifen in the ABCSG12 trial. The need for other predictive biomarkers to select appropriate therapy, nonetheless, remains real. Finally, attention to long-term benefits and side effects of therapy will continue to be vital for these young women. Dr Paul Goss from Boston, MA, USA, addressed the role of extended endocrine therapy in patients with ER-positive breast cancer. Despite frequent menopausal symptoms with either TAM or AIs, in the largest placebo-controlled AI trials in postmenopausal women (MA.17 and MA.P3), minimal changes in patient-reported QOL were reported. For extended TAM, rare, but life threatening, endometrial cancers and thromboemboli increase with longer durations of therapy. In contrast, AIs have not been associated with life-threatening side effects, but frequent mild bone loss, increasing the likelihood of osteoporosis, occurs although this can usually be overcome with concurrent use of oral bisphoshonate therapy, and BMD improves after cessation of AI therapy. Dr Ann Partridge from Dana-Farber Cancer Institute, Boston, MA, USA, focused on the management of very young patients with breast cancer. Breast cancer is the leading cause of cancer-related deaths in women who are aged 40 and younger in developed countries, and although generally improving, survival rates for young women with breast cancer remain lower than those for older women. Despite an increased risk of local recurrence, young age alone is not a contraindication to breast-conserving therapy, given the equivalent survival seen in this population with either mastectomy or breast conservation. Aggressive systemic therapy is often warranted for young women both from a chemotherapy and from a hormonal standpoint, given the substantial risk reduction. Multiagent chemotherapy and biologic therapy, targeting the tumour similar to the treatment in older women, are standard, with careful attention to unique survivorship concerns, including genetics, infertility and psychosocial concerns. Dr J Ingle, Mayo Clinic, Rochester, MN, USA, concluded the meeting with a discussion on the development of an optimal adjuvant endocrine therapy strategy for postmenopausal women and the requirement for a balance between potential benefits and potential toxicities of the therapeutic options. Toxicities can be considered in terms of serious adverse events (SAEs), which can be potentially life threatening, and adverse events (AEs) that are not life threatening per se, for example, musculoskeletal adverse events (MSAEs) and hot flashes, but which may represent a serious threat to a patient’s outcome (in terms of breast events) because of a negative impact on compliance. Several studies (ATAC, TEAM) have identified toxicities related to treatment (e.g. vasomotor, MSAEs) that are associated with outcome, but this association was not seen in MA.27. In postmenopausal women, the balance of benefit and toxicity favours the use of AIs in the adjuvant setting.

## Conclusion

The last decade has seen a remarkable technological explosion resulting in an entirely new field designated as ‘omics’. The application of high-throughput techniques for profiling DNA, RNA, and protein in breast cancer samples from hundreds of patients has profoundly increased our knowledge of breast cancer. However, many gaps in our knowledge remain, and these will require a long process of extended clinical correlation studies, deeper integrated ‘omic’ analysis and functional annotation to fill. The major issue that arose during the consensus conference is the increasing gap between what is theoretically feasible in patient stratification (genomic testing, next generation sequencing approach, disease segmentation) and treatment (dual targeting, integration of targeted agents) and the daily practice. This point was clearly and elegantly highlighted by Dr Angelo Di Leo during the consensus panel and was a common thread during the whole meeting. Can we really transfer into daily clinical practice the huge amount of ‘omics’ data? This is a major concern shared by all oncologists. Angelo Di Leo discussed it in regard to genomic tests for prognostic purpose, but this problem is broader and the general issue is, ‘access to innovation for patients with breast cancer: how to speed it up?’ The same limitations are related to clinical practice in the case of expensive drugs to be integrated in patient management (dual targeting in HER2-positive breast cancer). We really believe this is a major political issue: How to create access to innovation for citizens, bridging the gap between clinical trials and daily practice? In lots of European countries, many innovative therapies or diagnostic tests are not yet approved or reimbursed. We need to find a new path to access innovation that would translate clinical research into daily practice. Potential solutions for further improvement must entail new biology-driven approaches, since optimisation of conventional treatments has in many cases reached its limits. The introduction of drugs that are less toxic, more targeted and more expensive than those currently used necessitates a partnership between clinical and translational researchers, the pharmaceutical industry, drug regulators and patients and their families. This therapeutic alliance will ensure that efforts are focused on the unmet clinical needs of patients with breast cancer. The immediate priority for patients with breast cancer is to improve access to an affordable, best standard of care in every country. A strong link between quality of care and research activity is dependent on national resources and health-care priorities being aligned to support the improvement of care for patients with breast cancer. To ensure continued innovation that meets the needs of patients, the therapeutic alliance between patients and academic-led research needs to be extended to include relevant pharmaceutical companies and drug regulators. The other key issue around national planning is the increasing complexity of managing the rare and heterogeneous types of cancers with the increasing number of drugs specific for subpopulations. This difficulty creates the need for specialised centres of excellence and referral pathways as important components of care planning, increasing networking in national and continental healthcare programs. For access to innovation for breast cancer patients, we need to bring together major players from the world of breast cancer research to map out a coordinated strategy, on an international scale, to address this fragmentation. Many programs and investigators are pursuing their own separate paths in relative isolation and generating data without establishing how best to integrate their data with the larger research community. We need a coordinated, collaborative, communal effort to improve access to an affordable, best standard of care for every patient in every country.
